# Combination of trio-based whole exome sequencing and optical genome mapping reveals a cryptic balanced translocation that causes unbalanced chromosomal rearrangements in a family with multiple anomalies

**DOI:** 10.3389/fgene.2023.1248544

**Published:** 2023-09-07

**Authors:** Min Xie, Jiangyang Xue, Yuxin Zhang, Ying Zhou, Qi Yu, Haibo Li, Qiong Li

**Affiliations:** ^1^ The Central Laboratory of Birth Defects Prevention and Control, Ningbo Women and Children’s Hospital, Ningbo, Zhejiang, China; ^2^ Neonatal Screening Center, Ningbo Women and Children’s Hospital, Ningbo, Zhejiang, China

**Keywords:** case report, optical genome mapping, cryptic balanced translocation, unbalanced chromosomal rearrangements, multiple anomalies

## Abstract

**Background:** Balanced translocation (BT) carriers can produce imbalanced gametes and experience recurrent spontaneous abortions (RSAs) and even give birth to a child with complex chromosomal disorders. Here, we report a cryptic BT, t(5; 6) (p15.31; p25.1), in the proband’s grandmother, which caused unbalanced chromosomal rearrangements and various anomalies in the two subsequent generations. We also provide a thorough overview of the application of optical genome mapping (OGM) to identify chromosomal structural variants (SVs).

**Methods:** Trio-based whole exome sequencing (Trio-WES) was conducted to explore the genetic basis of the phenotype of the proband and her mother. High-resolution karyotype analysis and OGM detection were performed on the proband’s grandparents to trace the origin of the unbalanced rearrangements between chromosomes 5 and 6. A PubMed search was conducted with the following keywords: “OGM” and “SVs.” Then, relevant studies were collected and systematically reviewed.

**Results:** The proband and her mother presented with various anomalies, whereas the grandmother was healthy but had a history of four abnormal pregnancies. Trio-WES revealed a heterozygous duplication on the terminal region of chromosome 5p and a heterozygous deletion on the proximal end of chromosome 6p in the proband and her mother. High-resolution karyotype analysis revealed no aberrant karyotypes in either grandparent, whereas OGM detection revealed a cryptic BT, t(5; 6)(p15.31; p25.1), in the proband’s grandmother. An overwhelming majority of research publications have verified the clinical utility of OGM in detecting SVs.

**Conclusion:** The results of this study revealed that the unbalanced chromosomal rearrangements and many anomalies observed in multiple members of the family were attributable to the cryptic BT carried by the proband’s grandmother. This study supports that OGM has a unique advantage for detecting cryptic BTs, and can be used as a first-tier genetic test for the etiological diagnosis of infertility, RSAs, and other complex genetic disorders.

## 1 Introduction

Balanced translocations (BTs) are formed by the random *de novo* breakage and rejoining of two or more chromosomes and are classified as chromosomal structural rearrangements (Wang et al., 2020). It occurs in approximately 0.2% of the general population and 2.2% of patients with a history of recurrent spontaneous abortions (RSAs) (Jacobs et al., 1992; Ogilvie et al., 2001; Wilch and Morton, 2018). BT carriers can produce unbalanced gametes, resulting in recurrent implantation failure, early abortion, or fetal death (Chantot-Bastaraud et al., 2008; Hofherr et al., 2011; Wilch and Morton, 2018). Occasionally, a living fetus with an unbalanced chromosomal rearrangement may exhibit complex chromosome diseases. Keify et al. reported two novel familial BTs, t(8; 11)(p23; q21), and t(6; 16)(q26; p12), which are implicated in RSAs (Keify et al., 2012). Soltani et al. conducted cytogenetic studies on 608 couples with RSAs in northeastern Iran and found 25 BT cases (approximately 2.05%) (Soltani et al., 2021). Wan et al. have recently reported two rare Chinese Han cases of RSAs associated with chromosomal BTs (Wan et al., 2021).

Traditional karyotyping (KT) is the standard-of-care (SOC) genetic testing for large chromosome rearrangements; however, its relatively low resolution of 5–10 Mb G banding limits its use in identifying copy number variations (CNVs) and cryptic structural variants (SVs) (Trask, 2002; Wapner et al., 2012). Fluorescence *in situ* hybridization (FISH) is another efficient SOC approach that can precisely detect target SVs depending on the corresponding probes. However, it is not applicable for accurate analysis at the whole-genome level (Carpenter, 2001; Bates, 2011). Recently, next-generation sequencing (NGS) technologies, including whole-genome sequencing, have been applied to map chromosome breakpoints; however, this application sometimes may present technical difficulties in determining the accurate breakpoints of BTs (Liang et al., 2017; Nilsson et al., 2017; Hu et al., 2019).

More recently, optical genome mapping (OGM) technology, which is based on the analysis of ultra-high molecular weight (UHMW) DNA, allows the precise detection of chromosomal SV abnormalities. OGM can be used to directly label, straighten, linearize, and unfold each DNA molecule through very fine capillary electrophoresis, in which the segment of a complicated SV is easily spanned and mapped. Different from SOC testing, OGM exhibits a unique ability to detect nearly all classes of clinically significant SVs per sample, including aneuploidy, CNVs (deletions/duplications), balanced/unbalanced translocations, inversion, insertion, ring chromosomes, and the absence of heterozygosity (Sun et al., 2018; Mantere et al., 2021). A systematic literature review has summarized the clinical applications and performance evaluation of OGM in constitutional disorders and hematologic neoplasms. Furthermore, multiple studies have validated a nearly 100% concordance between OGM and SOC methods in different clinical settings.

In the present study, we uncovered a cryptic BT, t(5; 6)(p15.31; p25.1), in the proband’s grandmother through OGM analysis. This translocation was believed to underlie the unbalanced rearrangements between chromosomes 5 and 6 in the proband and her mother. Based on data from literature reviews, OGM can be used as a first-tier genetic test for detecting cryptic BTs.

### 1.1 Case description

**FIGURE 1:**
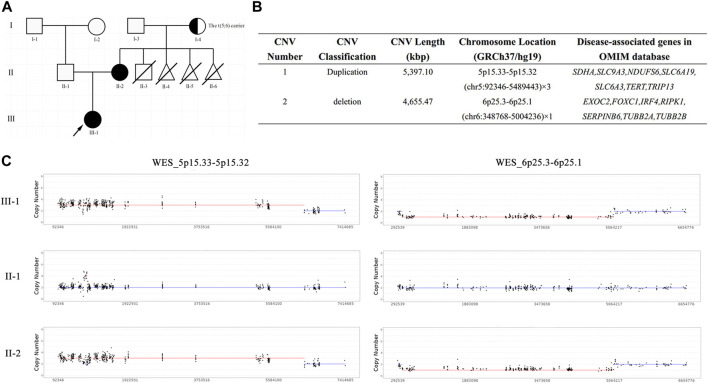
The pedigree chart and results of trio-WES. **(A)** The pedigree chart of this family: black represents the patients, arrow represents the proband, half-blackened circle represents the carrier of BT, oblique line represents the died; **(B)** The quantified CNVs of trio-WES for the proband and her mother; **(C)** The copy number of WES_5p15.33-5p15.32 and WES_6p25.3-6p25.1 in this family: III-1 represents the proband, II-1 represents the proband’s father, II-2 represents the proband’s mother.

The proband was an 8-year-old girl with multiple anomalies, including glaucoma, corneal opacities, deafness, tooth deformity, abnormal tongue, hypoglycemia, foot varus, and aberrant hip joint. She also presented with intellectual disability due to loss of language function and lack of response to normal communication, as well as a lag in growth, with initial standing at 3 years old and independent walking at 7 years old. The proband’s mother also showed several similar deformities but generally had milder phenotypes, including occasional nystagmus, pectus carinatum, low verbal skills, and intelligence. Notably, the proband’s grandmother was healthy but had experienced four abnormal pregnancies. Her first child died a month after birth, and the following three pregnancies were spontaneously aborted. The proband’s father, grandfather, and other grandparents showed no abnormalities. A pedigree chart of the family is shown in Figure 1A. The proband underwent eye and orthopedic surgeries prior to genetic testing. In addition, self-care rehabilitation was routinely conducted, but only slight symptomatic improvement was observed. The proband’s mother did not receive any therapeutic intervention. Written informed consent was obtained from all participants, and this study was approved by the ethics board of Ningbo Women and Children’s Hospital (No. EC 2020-048). This study conformed to the provisions of the Declaration of Helsinki as revised in 2013.

## 2 Methods

### 2.1 Trio-WES

Genomic DNA was isolated from the peripheral blood of the proband and her parents using a QIAamp DNA Blood Mini Kit (Qiagen, Valencia, CA, United States) in accordance with the manufacturer’s protocol. Trio-WES was performed using the HiSeq 2,500 sequencing platform (Illumina, San Diego, CA, United States). Raw fastq data (including CNVs) were filtered using fastp (v0.23.1) and annotated based on the human reference genome (hg19). BAM files were generated by re-ordering using Burrows–Wheeler Aligner (0.7.17-r1194-dirty). The Genome Analysis Toolkit (GATK, v4.20) was used to obtain single nucleotide polymorphisms (SNP) and insertions and deletions, and ANNOVAR and VEP were carried out for variation annotations. Pathogenicity classification and interpretation of sequence variants were performed in accordance with the guidelines of the American College of Medical Genetics and Genomics (ACMG). Sanger sequencing was performed to confirm the identified variants. CNVs were obtained using the eXome Hidden Markov model, and copy number was estimated using Lattice-Aligned Mixture Models (clamms). The pathogenicity classification of the identified CNVs was consistent with the consensus recommendations of ACMG and ClinGen in 2021 (Riggs et al., 2021).

### 2.2 High-resolution karyotype analysis

The Chromed CK cell synchronization kit (Shanghai Lechen Biotechnology Co. Ltd., Item No: CM-SK-80), which included high-resolution reagent solutions A (Fudr) and B (Thymidine), was used in this experiment. High-resolution karyotyping was performed in accordance with the manufacturer’s instructions. Briefly, 2 mL of anticoagulated peripheral blood (heparin) was collected from the proband’s grandparents. T lymphocytes in the peripheral blood were first stimulated into childish lymphocytes by plant lectins (PHA). After 72 h of cell culture, Solution A was added. After 16–18 h, the cells were treated with solution B. This process was maintained for 3–5 h, after which the cells were harvested using colchicine. After the experimental procedures of preparation, baking, drying, and sealing, the automatic scanning system GSL120 (Leica Biosystems Richmond, Inc., 5,205 Route 12 P. 0. Box 528 Richmond, Illinois 60071, United States) was used to automatically capture and record karyotypes. The karyotyping results were consistent with the *International Nomenclature System for Human Cytogenetics* (ISCN 2020).

### 2.3 OGM

OGM experiments were performed in accordance with the manufacturer’s instructions. Briefly, 2 mL of anticoagulant peripheral blood (EDTA) was collected from the proband’s grandparents. UHMW gDNA was extracted using the Blood and Cell DNA Isolation Kit v2 (Bionano Genomics, San Diego, CA, United States). Then, UHMW gDNA molecules were labeled with the DLS (Direct Label and Stain) DNA Labeling Kit (Bionano Genomics, San Diego, CA, United States of America). The DNA was quantified using a Qubit dsDNA Assay BR Kit with a Qubit 3.0 Fluorometer (Thermo Fisher Scientific). The labeled UHMW gDNA was loaded onto a Saphyr chip for linearization and imaged using a Saphyr instrument (Bionano Genomics, San Diego, CA, United States). Bionano Access v1.7 software was used for bioinformatics analysis, which included *de novo* assembly and comparison with a reference genome map (hg19).

### 2.4 Literature review

A PubMed search was performed using the following keywords: “OGM” and “SVs”. Studies concerning the clinical application of OGM for detecting chromosomal SVs in human samples were downloaded and systematically reviewed. Detailed information, including the aim of clinical research, results and clinical efficacy evaluation, publication date, and PMID, is summarized in Table 1.

**TABLE 1:** The systematical overview of OGM application and clinical utility for detecting chromosomal SVs.

No.	The aim of clinical research	Results and clinical efficacy evaluation	Publication date	PMID
1**	Comprehensive discernment of the complex and disperse mutational spectrum of solid tumor oligodendroglioma by OGM.	Over a thousand structural variants were identified in each tumor sample, and often in genomic regions deemed intractable by other technologies. The OGM system provides a rich description of the complex genomes of solid tumors	2013 July 26	23885787
2**	This study presented the application of optical mapping to discover new structural variants in a primary MM genome	This study characterized widespread structural variation in this tumor genome, and demonstrated an increase in mutational burden with tumor progression at all length scales of variation	2015 June 23	26056298
3**	This study presented optical mapping data for two human genomes: the HapMap cell line GM12878 and the colorectal cancer cell line HCT116	Their optical mapping data provide a resource for genome structure analyses of the human HapMap reference cell line GM12878, and the colorectal cancer cell line HCT116	2015 December 29	26719794
4	Analyzing optical genome maps of 154 individuals from the 26 populations sequenced in the 1000 Genomes Project	This study find that phylogenetic population patterns of large SVs are similar to those of single nucleotide variations in 86% of the human genome, while ∼2% of the genome has high structural complexity	2019 March 4	30833565
5	This study used optical mapping in nanochannel arrays for 154 human genomes from 26 populations to present a comprehensive look at human subtelomere structure and variation	This study discovered novel subtelomeric structural variants, resolution of sequence gaps and delineation of long-range subtelomeric haplotypes across 154 genomes	2020 January 27	31986135
6#	Performing rapid prenatal diagnoses of FSHD1 using a combination of Bianano optical mapping and linkage-based karyomapping	Bianano optical mapping can determine the number of D4Z4 repeats and exclude interference of the 10q26.3 homologous region, and in combination with karyomapping, can be used for rapid and accurate prenatal diagnosis of FSHD1	2020 Feb	31711258
7+	SMOM was applied to ascertain whether the BRT disrupted any genes associated with normal fertility	The SMOM has potential clinical application as a rapid tool to screen patients with BRTs for underlying genetic causes of infertility and other diseases	2020 Mar	32026199
8	Uncover potential risk factors for germline rearrangements leading to 22q11.2DS offspring	Analysis of deletion breakpoints indicates that preferred recombinations occur between FAM230 and specifc segmental duplication orientations within LCR22A and LCR22D, ultimately leading to NAHR.	2020 July 22	32699385
9	Chromosomal karyotyping, bionano OGM and CNV-seq were used to delineate the chromosomal aberration carried by a patient	Bionano OGM has provided a novel tool for the detection and diagnosis of structural chromosomal aberrations	2020 October 10	32924127
10	Whole-genome sequencing and whole-genome optical mapping, complementary next-generation genomic technologies were used for the nonverbal proband and his mother	These technologies were capable of the accurate and robust detection of structural variants, identified t(3; 10), t(10; 14), and t(3; 14) three-way balanced translocations in the mother and der(10)t(3; 14; 10) and der(14)t(3; 14; 10) translocations in the patient	2020 December 17	33335013
11	This study characterized SDs and identified novel SVs at 7q11.23, 15q13.3, and 16p12.2 using OGM data	This study detected several novel SVs for each locus. The OGM will greatly facilitate the investigation of the role of inter-SD structural variation as a driver of chromosomal rearrangements and genomic disorders	2021 February 9	33724415
12	By using OGM and long-read sequencing, this study aimed to identify the pathogenic variant in a large family with X-linked choroideremia	This study revealed an intragenic 1,752 bp inverted duplication, located downstream of the wild-type copy of exon 12. OGM and long-read sequencing have significant potential for the identification of (hidden) structural variants in rare genetic diseases	2021 July 20	35047838
13#	This commentary highlights the potential for OGM to become a standard of care in prenatal genetic testing	OGM can provide a high-resolution, cytogenomic assay to be employed following a positive NIPT screen or for high-risk pregnancies with an abnormal ultrasound	2021 March 11	33799648
14	This proof-of-principle study demonstrates the ability of OGM to detect nearly all types of chromosomal aberrations	OGM reached 100% concordance compared to standard assays for all aberrations with non-centromeric breakpoints	2021 August 5	34237280
15*	Samples from 52 individuals with a clinical diagnosis of a hematological malignancy, divided into simple and complex cases, were processed for OGM.	For the 36 simple cases, all clinically reported aberrations were detected. For the 16 complex cases, results were largely concordant between standard-of-care and OGM, but OGM often revealed higher complexity than previously recognized	2021 August 5	34237281
16	Short-read whole-genome sequencing data and OGM were performed for a 5-yearold female with a constellation of clinical features consistent with CSS1	This study revealed additional inversions, all clustered on chromosome 6, one of them disrupting ARID1B for which haploinsufficiency leads to the CSS1 disease trait	2021 August 26	34512724
17*	Twelve pediatric ALL samples were analyzed by OGM, and results were validated by comparing OGM data to results obtained from routine diagnostics	All genomic aberrations known from other techniques were also detected by OGM. OGM was superior to well-established techniques for resolution of the more complex structure	2021 August 30	34503197
18	Whole exome and whole genome sequencing failed to identify germline or somatic SMARCB1 pathogenic mutations, and OGM was performed as an alternative strategy to identify structural variation in this locus	An insertion of 2.8 kb within intron 2 of SMARCB1 was detected. This case demonstrates the power of OGM to identify genomic variations that are refractory to detection with standard techniques	2021 Oct	34231212
19*	This study compared OGM to standard techniques in 10 selected B or T-ALL.	Eighty abnormalities were found using standard techniques of which 90% were correctly detected using OGM. OGM represents a promising alternative to cytogenetic techniques currently performed for ALL characterization	2021 Oct	33982372
20	To confirm a total of 14 different structural or numerical chromosomal variants originally detected by other means	12 variants could be confirmed. This study tested the successful application of OGM in routine diagnostics and described some of the challenges	2021 December 8	34946907
21	To identify rare/unique SVs as decisive predisposition factors associated with COVID-19	This study is the first to systematically assess the potential role of SVs in the pathogenesis of COVID-19 severity	2022 February 18	35036860
22	Ten cases with abnormal karyotype was detected by OGM.	Nine were detected with abnormality by OGM. OGM can both detect unbalanced rearrangements, balanced translocations and inversions. It can refine breakpoints and orientation of duplicated segments or insertions	2022 May 6	35644979
23	10 genetic outpatients were detected simultaneously by karyotype analysis, FISH, CNV-seq, and Bionano OGM.	OGM allowed the location of the mutation to the gene level, can be a high adjunctive diagnostic method for detecting balanced chromosome translocations	2022 Jun	35384386
24+	Eleven couples with normal karyotypes that had abortions/affected offspring with unbalanced rearrangements were enrolled for OGM. Other technologies were implemented to confirm the results obtained from OGM.	High-resolution OGM successfully detected cryptic reciprocal translocation in all recruited couples, which was consistent with the results of FISH and sequencing	2022 June 16	35710108
25*	Review the strengths and weaknesses of OGM compared to standard of care techniques and illustrate how the technique is likely to change front line testing in many hematologic malignancies	Parallel studies of OGM versus standard of care testing have demonstrated it can detect and resolve more abnormalities than karyotyping or FISH.	2022 Jul	35560245
26	This study used amplification-free targeted sequencing and OGM to decipher the composition of these repeat expansions	Long-read sequencing and OGM of repeat expansions are critical for clinical practice and genetic counseling	2022 August 15	36092952
27#	Applying a combination of genetic methods for a pregnant woman, who had a spontaneous abortion last year, had abnormal prenatal test results again in the second pregnancy	The pregnant woman was a carrier of a CCR involving three chromosomes and four breakpoints. Her frst and second pregnancy abnormalities were caused by chromosomal microdeletions and microduplications	2022 September 21	36127686
28**	This study tested the feasibility of OGM to detect complex genomic rearrangements in consecutive unselected PCa samples from MRI/US-fusion targeted biopsy	Clinically relevant genomic SVs were successfully detected in PCa samples by OGM.	2022 October 8	36209207
29*	This study reported the results from their clinical validation study and demonstrate the utility of OGM.	OGM has outperformed the standard-of-care tests in this study and demonstrated its potential as a first-tier cytogenomic test for hematologic malignancies	2022 Dec	36265723
30*	This study utilized OGM to determine chromosomal aberrations in 46 children with BALL and compared the results of OGM with conventional technologies	OGM showed a good concordance with conventional cytogenetic techniques in identifying the reproducible and pathologically significant genomic SVs. OGM has a greater ability to detect complex chromosomal aberrations, refine complicated karyotypes, and identify more SVs	2022 December 21	36612032
31	This study combined advanced bioinformatics screening of 3000 genomes and 1,500 exomes with OGM and long-read-sequencing for confirmation studies	The latest state-of-the-art technologies (OGM, long-read-sequencing) effectively flag complex repeat expansions using short read datasets and thus facilitate diagnosis of ultra-rare disorders	2023 January 1	35913761
32	The preparation of samples and subsequent loading and analysis in an OGM system is discussed for the detection and analysis of FSHD.	These methods should prove highly useful in FSHD analyses and beyond with the development of further disease analysis pipelines within the instrument	2023 Jan	36648278
33*	This study reported the results evaluating the concordance of OGM and standard-of-care FISH in 18 CLL samples	The results were fully concordant between these two techniques in the blinded comparison. OGM revealed additional aberrations in 78% of the samples, and enabled the detection of complex karyotypes that were undetectable by FISH in three samples	2023 February 17	36831635
34*	This review summarized the current knowledge of OGM with regard to diagnostics in hematological malignancies, and AML in specific	This review focuses on the ability of OGM to expand the use of cytogenetic diagnostics in AML and perhaps even replace older techniques	2023 March 9	36980569
35	OGM was performed to improve SV detection in short-read genome sequencing-negative cases	OGM identified a pericentric inversion (173 megabase), with 1 breakpoint disrupting USH2A	2023 Mar	36524988
36	OGM results of 50 negative controls and 359 samples from individuals with suspected genetic conditions were compared with SOC for technical concordance, clinical classification concordance, intrasite and intersite reproducibility, and ability to provide additional, clinically relevant information	Technical concordance for OGM to detect previously reported SOC results was 99.5%. The blinded analysis and variant classification agreement between SOC and OGM was 97.6%. Replicate analysis of 130 structural variations was 100% concordant	2023 Mar	36828597
37*	11 adult ALL patients were examined using OGM. Genetic results obtained by karyotyping and FISH were confirmed by OGM for all patients	Additional genetic information was obtained in 82% of samples by OGM, previously not diagnosed by standard of care	2023 March 9	36980958
38	OGM was performed to identify SVs from 87 individuals with MRKH and available parents, which were confirmed by another method when possible	Thirty-four SVs that overlapped coding regions of genes with potential involvement in MRKH were identified, 14 of which were confirmed by a second method	2023 Apr	36797380
39*	This study reported the performance of OGM in a cohort of 100 AML cases that were previously characterized by karyotype alone or karyotype and FISH or CMA.	OGM identified all clinically relevant SVs and CNVs reported by these standard cytogenetic methods, as well as clinically relevant information in 13% of cases that had been missed by the routine methods	2023 April 11	36417763
40*	The results obtained using OGM and standard techniques were compared in 29 cases of AML or ALL.	OGM detected 73% of abnormalities identified by standard methods, and identified additional abnormalities in six cases. OGM is an attractive alternative to current multiple cytogenetic testing in acute leukemia	2023 April 3	37046792
41#	This validation study analyzed 114 samples for traditional cytogenetic analysis with karyotyping, FISH, and/or CMA.	OGM was 100% concordant in identifying the 101 aberrations, and detected 64 additional clinically reportable SVs in 43 samples. This study demonstrated the potential of OGM to replace the current SOC methods for prenatal diagnostic testing	2023 Apr	36758723

**Notes:** * Represents the application of OGM, in hematologic neoplasms; ** Represents the application of OGM, in other solid tumors; # Represents the application of OGM, in prenatal disgnosis; + Represents the application of OGM, in couples with infertility or RSAs.

**Abbreviations:** SDs, Segmental duplications; BRT, balanced reciprocal translocation; SMOM, single-molecule optical mapping; FISH, fluorescence *in situ* hybridization; CNV-seq, copy number variation sequencing; NAHR, non-allelic homologous recombination; CCR, complex chromosomal rearrangement; CSS1, Coffin–Siris syndrome 1; MM, multiple myeloma; PCa, prostate cancer; GLS, glutaminase; ALL, acute lymphoblastic leukemia; BALL, B-cell Acute lymphoblastic leukemia; FSHD1, facioscapulohumeral muscular dystrophy 1; MRKH, Mayer-Rokitansky-Kuster-Hauser; AML, acute myeloid.

## 3 Results

### 3.1 Unbalanced rearrangements between chromosomes 5 and 6 were discovered in proband and her mother

To unveil the genetic basis of the phenotype and ascertain the clinical diagnosis, we first performed trio-WES on the proband and her parents. The proband carried a heterozygous duplication of 5,397.10 Kbp in the 5p15.33–5p15.32 region of chromosome 5 and a heterozygous deletion of 4,655.47 Kbp in the 6p25.3–6p25.1 region of chromosome 6 (Figures 1B, C), both of which were inherited from her mother. These unbalanced rearrangements were not detected in the father of the proband (Figure 1C). A duplication in chromosome 5, which is a non-polymorphic variant, was not reported in the DGV database. The DECIPHER database described a similar case with clinical phenotypes including cerebral hemorrhage, hearing impairment, neurodevelopmental delay, and strabismus (Patient ID: 339222). In accordance with the ACMG guidelines, the proband’s duplication was classified as having unknown clinical significance.

The deletion on chromosome 6 contained *FOXC1* with a haploinsufficiency score of 3. Heterozygous mutation in *FOXC1* could lead to Anterior segment dysgenesis 3 (MIM number: 601631) and Axenfeld–Rieger syndrome type 3 (RIEG3, MIM number: 602482). ASGD and ASMD are heterogeneous developmental disorders that affect the anterior segment of the eye, including the cornea, iris, lens, trabecular meshwork, and Schlemm’s canal. The clinical features of ASGD include iris hypoplasia, enlarged or reduced corneal diameter, corneal vascularization and opacity, posterior embryotoxin, corectopia, polycoria, abnormal iridocorneal angle, ectopia lentis, and anterior synechiae between the iris and posterior corneal surface (Cheong et al., 2016). The clinical phenotypes of RIEG3 are highly heterogeneous and include zygomatic planar process, ocular hypertelorism, sensorineural deafness, glaucoma, eyeball protrusion, posterior embryonic ring, denture, microdentin, vermium hypoplasia, atrial septal defect, patent ductus arteriosus, iris hypoplasia, pupil dislocation, concave nasal ridge, and midface retroretraction (Gould et al., 1997; Cunningham et al., 1998). The deletion in the 6p25.3–6p25.1 region, which is a non-polymorphic variant, was not included in the DGV database. Conversely, the DECIPHER database enrolled a crowd of cases presenting with multiple anomalies and carrying similar pathogenic/likely pathogenic CNVs (Patient ID:308608, 332098, 401710, etc.). Combined with the proband’s clinical symptoms, the deletion in the 6p25.3–6p25.1 region was classified as pathogenic.

### 3.2 Cryptic BT t(5; 6) was revealed in the proband’s grandmother

**FIGURE 2:**
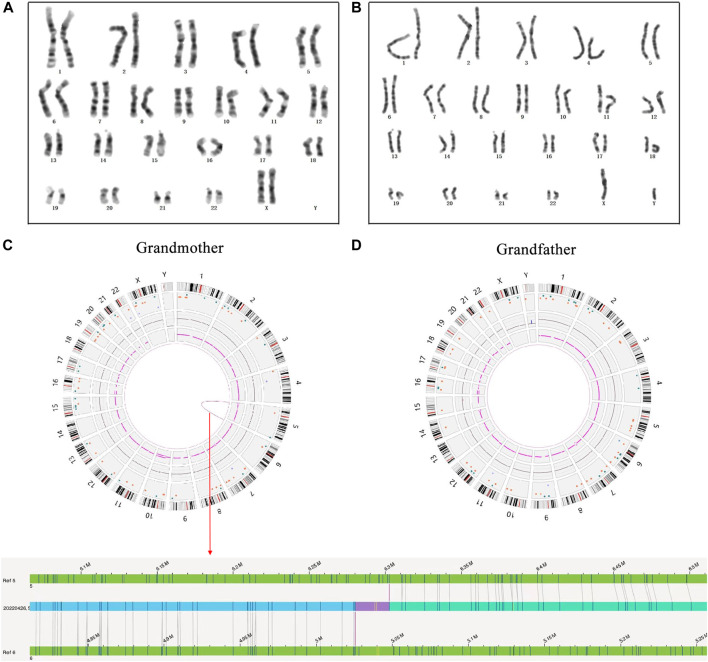
The results of high-resolution karyotype analysis and OGM analysis for the proband’s grandparents. **(A)** The description of grandmother’s karyotype was shown as 46,XX; **(B)** The description of grandfather’s karyotype was shown as 46,XY; **(C)** A cryptic BT between chromosomes 5 and 6 was uncovered in the grandmother by OGM: red arrow represents OGM analysis result; **(D)** No abnormity was found in the grandfather by OGM.

**FIGURE 3:**
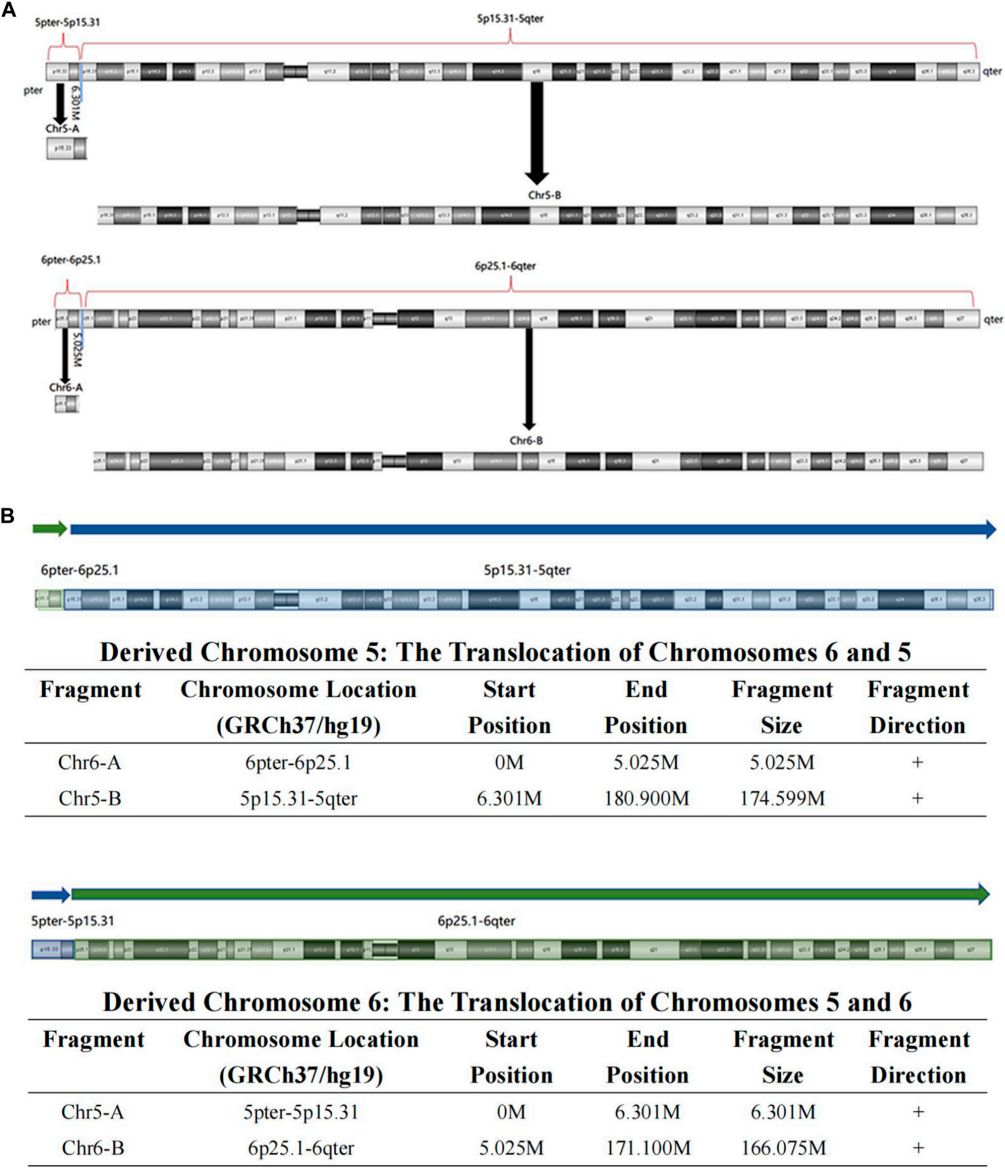
The visual BT results of OGM in the grandmother. **(A)** According to the breakpoint, the whole chromosome 5 was separated into Chr5-A and Chr5-B, and the whole chromosome 6 was separated into Chr6-A and Chr6-B; **(B)** The derived chromosome 5 comprises Chr6-A (6pter-6p25.1, 5.025 M) and Chr5-B (5p15.31-5qter, 174.599 M), and the derived chromosome 6 comprises Chr5-A (5pter-5p15.31, 6.301 M) and Chr6-B (6p25.1-6qter, 166.075 M).

High-resolution karyotype analysis was performed to trace the origin of the unbalanced rearrangements between chromosomes 5 and 6 in the proband’s grandparents. However, the grandmother and grandfather both exhibited normal karyotypes (Figures 2A, B). Given that OGM has a 10,000-fold higher resolution than karyotyping, the grandparents’ peripheral blood was further subjected to OGM. As expected, no abnormalities were found in the grandfather (Figure 2D), whereas a cryptic BT between chromosomes 5 and 6 was uncovered in the grandmother (Figure 2C), which may explain the 5.4 Mb 5p terminal duplication and 4.7 Mb 6p terminal deletion in the proband and her mother. According to the breakpoints on the chromosomes, chromosome 5 was separated into Chr5-A (5 pter-5p15.31, 6.301 M) and Chr5-B (5p15.31-5 qter, 174.599 M), and chromosome 6 was separated into Chr6-A (6 pter-6p25.1, 5.025 M) and Chr6-B (6p25.1-6 qter, 166.075 M) (Figure 3A). The derived chromosome 5 comprised Chr6-A and Chr5-B, and the derived chromosome 6 comprised Chr5-A and Chr6-B (Figure 3B).

### 3.3 Literature review of OGM application and clinical utility for detecting chromosomal SVs

Most studies have validated a 100% clinical concordance of OGM with traditional cytogenetic analysis and identified additional cryptic variations that remain beyond the purview of current technologies (Table 1). OGM has been extensively used to characterize SVs for the diagnosis of hematological neoplasms. The study by Luhmann et al. (2021) indicated that 12 pediatric acute lymphoblastic leukemia samples were analyzed using OGM, and all genomic aberrations identified by other techniques were also detected using OGM. Their results showed that OGM was superior to well-established techniques for the resolution of complex structures and had a higher sensitivity for the detection of copy number alterations (Luhmann et al., 2021). Sahajpal & Mondal et al. also demonstrated that OGM outperforms SOC tests and may be a first-tier cytogenomic test for hematologic malignancies (Sahajpal et al., 2022b). Recently, Levy’s group reported in a real-world setting the performance of OGM in a cohort of 100 acute myeloid leukemia (AML) cases that were previously characterized by KT alone or KT and FISH or CMA. Their multicenter study indicates that OGM effectively recovers clinically relevant SVs and CNVs found by SOC methods and reveals an additional 13% of cases that had been missed by the routine methods (Levy et al., 2023). Moreover, the application and clinical utility of OGM have been reported in other solid neoplasms, including multiple myeloma (Gupta et al., 2015), solid tumor oligodendroglioma (Ray et al., 2013), and prostate cancer (Shim et al., 2022).

Aside from its superiority in the precise diagnosis of oncology, OGM also has greater efficiency in detecting postnatal/prenatal constitutional genetic disorders. A previous multi-site assessment of OGM in constitutional postnatal cases indicated that the technical concordance for OGM to detect previously reported SOC results was 99.5%, and the replicate analysis of 130 SVs was 100% concordant (Iqbal et al., 2023). Additionally, high-resolution OGM has been successfully used to detect cryptic reciprocal translocation in 11 couples with normal karyotypes and abortions/affected offspring with unbalanced rearrangements (Zhang et al., 2022). Another study used a combination of OGM and linkage-based karyomapping for the rapid prenatal diagnoses of facioscapulohumeral muscular dystrophy 1 (Zheng et al., 2020). A commentary by Sahajpal et al. in 2021 highlighted the potential of OGM as a standard of care in prenatal genetic testing (Sahajpal et al., 2021). Furthermore, Sahajpal & Jill et al. performed OGM in 52 patients with severe COVID-19 to identify rare/unique SVs as decisive predisposition factors associated with COVID-19. This study is the first to systematically assess the potential role of SVs in the pathogenesis of COVID-19 severity (Sahajpal et al., 2022a). Systematic information on OGM application and clinical utility for detecting chromosomal SVs was collected and summarized in Table 1.

## 4 Discussion

Trio-WES has been widely used as a first-tier tool for the molecular diagnosis of rare genetic diseases. Along with improvements in CNV algorithms using WES data, WES, especially trio-WES, is already widely accepted as a cost-effective diagnostic assay for rare genetic disorders resulting from pathogenic CNV (Zhai et al., 2021). In this case, trio-WES indicated an unbalanced rearrangement between chromosomes 5 and 6 in the proband and her mother. Given the grandmother’s four abnormal pregnancies, we speculated a potential BT in one of the proband’s grandparents.

Conventional KT has a limited resolution in the recognition of small or cryptic SVs. Our case report again demonstrated its limited usage in this regard because the proband’s grandparents had normal results on high-resolution KT analysis. However, the genetic basis of more than half of these patients remains unclear even though multiple tests, including KT, CMA, and NGS, are used in clinical settings. OGM is widely accepted as a novel tool for detecting SVs. Several factors, including cost, high sample requirement, and the absence of a standard data analysis pipeline, limit its clinical application at this stage. Nevertheless, an increasing number of clinical studies have shown that OGM has superior performance in the detection of SVs compared to SOC assays (e.g., KT, FISH, and CMA) (Table 1). In the present study, OGM analysis showed that the grandmother carried a cryptic BT between chromosomes 5 and 6, which revealed the origin of the unbalanced rearrangements in the proband and her mother. The findings of this study particularly emphasized OGM’s unique advantage for detecting cryptic BTs.

We also reviewed the applications and clinical utility of OGM for detecting chromosomal SVs. OGM has been thoroughly evaluated for potential clinical applications in postnatal and prenatal scenarios. A vast majority of studies have demonstrated the utility of OGM in detecting nearly all types of chromosomal aberrations and highlighted its clinical superiority to SOC assays in detecting complex cryptic SVs. In assisted reproduction, OGM has been used to clarify the etiology of infertility, recurrent spontaneous abortion, and abnormal pregnancy histories (Wang et al., 2020; Yang and Hao, 2022; Zhang et al., 2022). This technology not only helps optimize reproductive decisions but also avoids the birth of children with severe genetic diseases. Certainly, if this cryptic BT t(5; 6) of the grandmother in our case report had been uncovered earlier, her repeated aberrant pregnancy history and multiple anomalies of the two subsequent generations could have been effectively avoided through preimplantation genetic testing.

In addition, the methodology and algorithm of OGM have been researched and optimized to facilitate its utility in detecting complex SVs. In 2021, Mukherjee et al. extended the definition of bilabels in the paired de Bruijn graph to the context of optical mapping data and presented the first de Bruijn graph-based method for Rmap assembly. This method has been successfully applied to three genomes: *E. coli*, human, and climbing perch fish (Anabas Testudineus) (Mukherjee et al., 2021). Other tools, such as OMTools (Leung et al., 2017) and OMSV (Li et al., 2017), have also been developed to assist in optical mapping analysis. With technological developments and growing applications, OGM is expected to enable the assembly of more complete genomes and discovery of novel variations in the near future.

Overall, our case report and literature review support the indispensable role of OGM in identifying genomic cryptic BTs, that SOC tests could not find out. The limitation of this study is the absence of long-term follow-up for this special family and up-to-date literature summary, which may warrant further discussion.

## 5 Conclusion

In this case report, the combined use of Trio-WES and OGM revealed an unbalanced rearrangement between chromosomes 5 and 6 in the proband and her mother, as well as a cryptic BT t(5; 6)(p15.31; p25.1) in the proband’s grandmother, which clarified the genetic etiology of the grandmother’s abnormal fertility history and multiple anomalies in the two subsequent generations. Our systematic literature review demonstrates the utility of OGM in detecting and diagnosing chromosomal SVs. Collectively, our study summarized that OGM has an inherent advantage for identifying cryptic BTs, especially when combination with trio-WES or other SOC tests, could provide precise etiological diagnosis of couples with infertility, RSAs, or other patients with solid tumors and other complicated diseases.

## Data Availability

The datasets for this article are not publicly available due to concerns regarding participant/patient anonymity. Requests to access the datasets should be directed to the corresponding authors.
